# Silencing of Human Phosphatidylethanolamine-Binding Protein 4 Enhances Rituximab-Induced Death and Chemosensitization in B-Cell Lymphoma

**DOI:** 10.1371/journal.pone.0056829

**Published:** 2013-02-25

**Authors:** Kai Wang, Yu Jiang, Weiyan Zheng, Zhiyong Liu, Hui Li, Jianzhou Lou, Meidi Gu, Xiaojian Wang

**Affiliations:** 1 Department of Respiratory Medicine, The Second Affiliated Hospital, School of Medicine, Zhejiang University, Hangzhou, People’s Republic of China; 2 Institute of Immunology, School of Medicine, Zhejiang University, Hangzhou, People’s Republic of China; 3 Department of Hematology, First Affiliated Hospital, Zhejiang University, School of Medicine, Hangzhou, Zhejiang, People’s Republic of China; University of North Carolina at Chapel Hill, United States of America

## Abstract

Rituximab is the first line drug to treat non Hodgkin’s lymphoma (B-NHL) alone or in combination with chemotherapy. However, 30–40% of B-NHL patients are unresponsive to rituximab or resistant after therapy. Human phosphatidylethanolamine-binding protein 4 (hPEBP4) is a novel member of PEBP family and functions as an anti-apoptotic molecule. In this study, we found hPEBP4 to be expressed in up to 90% of B-cell lymphoma patients, but in only 16.7% of normal lymph nodes. Interestingly, hPEBP4 overexpression inhibited rituximab-mediated complement dependent cytotoxicity (R-CDC) and antibody-dependent cell-mediated cytotoxicity (ADCC) in B-NHL cells while downregulation of hPEBP4 augmented the therapeutic efficacy of rituximab both *in vitro* and *in vivo*. Furthermore, hPEBP4 silencing sensitized the primary B-acute lymphocytic leukemia (B-ALL) cells to R-CDC. During rituximab-mediated complement dependent cytotoxicity, hPEBP4 was recruited to the cell membrane in a PE-binding domain dependent manner and inhibited R-CDC induced calcium flux and reactive oxygen species (ROS) generation. These events contributed to the decrease of cell death induced by R-CDC in B-cell lymphomas. Meanwhile, hPEBP4 knockdown potentiated the chemosensitization of the rituximab in B-cell lymphoma cells by regulating the expression of Bcl-xl, Cycline E, p21^waf/cip1^ and p53 and the activation of caspase-3 and caspase-9. Considering that hPEBP4 conferred cellular resistance to rituximab treatment and was preferentially expressed in lymphoma tissue, it could be a potential valuable target for adjuvant therapy for B-cell lymphoma.

## Introduction

Non-Hodgkin’s lymphoma (NHL) comprises a large group of neoplasms of the immune system and represents a heterogeneous group of diseases characterized by the monoclonal expansion of either B- or T- lymphocytes. The majority of NHL originates from B cells and represents a subpopulation of pre-B cells and a few mature B cells [1]. Rituximab (anti-CD20 monoclonal antibody) has been an important addition to the therapeutic arsenal against B-NHL. Since being approved for clinical use in 1997, rituximab has benefited many B-cell lymphoma patients, and has changed the treatment regimen in indolent lymphomas [2], [3]. The mechanisms suspected to mediate the therapeutic effects of rituximab include complement-dependent cytotoxicity (CDC), antibody-dependent cell-mediated cytotoxicity (ADCC) and chemosensitization [4], [5]. CDC involves the formation of the membrane attack complex (MAC) and activation of caspase-independent cell death involving ROS generation [6]. In ADCC, rituximab engages the Fc receptors on immune effector cells, stimulating the effector function of immune cells to induce target cell death. However, a subset of NHL patients relapse and are essentially considered incurable [7]. Meanwhile, rituximab resistance has emerged as an important clinical challenge, in that a subset of NHL patients does not respond to rituximab despite expressing CD20 [8]. A better understanding of anti-CD20 mAbs rituximab resistance mechanisms will support to develop new and more effective therapeutic approaches. Many researchers have made substantial efforts to address this issue. Low CD20 expression levels [9], [10], the presence of complement inactivating molecules(CD55,CD59) [6], [11], soluble CD20, shaving and downmodulation of CD20 [4], contribute to the resistance of lymphoma to rituximab therapy. Another study by Jazirehi et al showed that the rituximab-resistant clone exhibits hyperactivation of the P38, ERK1/2 MAPK and NF-κB pathways and high expression of Bcl-2/Bcl-xl [12], [13].

Human phosphatidylethanolamine-binding protein 4(hPEBP4) is a novel member of the PEBP family and functions as an anti-apoptotic molecule. hPEBP4 is preferentially expressed in several cancers, including breast cancer, prostate cancer, lung squamous cell carcinoma and colorectal cancer [14]–[19]. Silencing of hPEBP4 sensitizes the cancer cells to TNF-α/tumor necrosis factor-related apoptosis-inducing ligand (TRAIL)-induced apoptosis by increasing activation of JNK (c-Jun N-terminal kinase) and the Raf-1/MEK/ERK pathway, indicating that hPEBP4 is a candidate target molecule [14]–[17]. Recently, Qiu et al have indentified IOI-42 as the first chemical inhibitor of hPEBP4 and have shown that IOI-42 can sensitize tumor cells to TNF-α and TRAIL-mediated apoptosis by targeting hPEBP4 [20]. However, it is almost impossible to expect that TNF-α or TRAIL can accumulate to a concentration high enough to kill tumor cells *in vivo*, let alone determine the relationship of TNF-α level in patients and prognosis. Therefore, the exact role of hPEBP4 in cancer therapy remains unknown. Here, we found hPEBP4 to be highly expressed in human lymphoma samples. Since rituximab is being used currently in B-NHL treatment, we aimed to determine whether hPEBP4 could be a candidate target to increase the rituximab efficacy in B-NHL patients. hPEBP4 not only inhibited rituximab-mediated ADCC and CDC in B-NHL cells, but also limited its chemosensitization effect. Furthermore, we demonstrated that hPEBP4 represses rituximab-mediated CDC effect via inhibiting calcium flux and reactive oxygen species (ROS) production. Considering the B-NHL cells and primary B-ALL cells that have down regulated hPEBP4 were more sensitive to rituximab-induced death, hPEBP4 may be a new target for adjuvant therapy for B-NHL patients and that downregulation of hPEBP4 might represent a promising adjuvant regimen along with rituximab for B-cell lymphoma treatment.

## Materials and Methods

### Patient Samples

B-ALL cells were obtained from peripheral blood of diagnostic patients at the Department of Hematology, First Affiliated Hospital, Zhejiang University, PR China and enriched by density centrifugation over Lymphocytes Separation Medium (PAA Laboratories, Cölbe, Germany) [21]. The purity of the malignant cells was measured by CD20 staining. B-ALL was classified according to French-American-British (FAB) and World Health Organization (WHO) guidelines [22], [23]. Written informed consent and Institutional Review Board approval were obtained for all the samples. Experiments using the patient samples were approved by the Zhejiang University ethics committee. (Permit Number: 2011–11).

### Reagents and Plasmid

Rituximab was obtained from Roche Ltd (Hoffmann-La Roche Ltd. Basel Switzerland). Camptothecin(CPT), a cytotoxic quinoline alkaloid, causes DNA damage which results in cell apoptosis [24], was purchased from Sigma (Sigma, St. Louis, MO). Anti-phospho ERK1/2, anti-phosphop 38, anti-phospho Syk antibodies were obtained from Cell Signaling Technology (Cell Signaling Technology, Beverly, MA, USA). Antibodies specific to Actin, Bcl-xl, Bcl2, Bim, Bak, Bad, Bax, Bag-1, procaspase-3, procaspase-8, prcaspase-9,p53, p21^waf/cip1^ were from Epitomics (Epitomics, Burlingame, CA USA). hPEBP4 antibody was purchased from Abcam (Abcam Inc., Cambridge, MA, USA). DyLight647 conjugated anti-goat IgG (Rockland Inc, Gilbertsville, PA, USA) was used as secondary antibody. ROS Fluorescent Probe-DHE was obtained from Vigorous (Vigorous, Beijing, China). Plasmid pDsRed-mem, Fluo-4 and the non-ionic detergent Pluronic F-127 were obtained from Invitrogen (Invitrogen Carlsbad, CA USA).

### Plasmid and siRNA Transfection

21-nucleotide sequences of hPEBP4 siRNA were synthesized by Proligo: 5-GGAAAAGUCAUCUCUCUCCTT (sense) and 5′-GGAGAGAGAUGACUUUUCCTT (antisense). hPEBP4 mutation control siRNA oligonucleotides were 5′-GGAAAAUCUACUCUCUCCTT (sense) and 5′-GGAGAGAGUAGACUUUUCCTT (antisense). hPEBP4 transient siRNA assay with chemically synthesized siRNA duplex and mutated control was done as previously described [15]. For stable silencing of hPEBP4 expression, Raji and Daudi B-NHL cells were transfected with the hPEBP4-RNAi or shNC plasmid () using the Amaxa nucleofector (Amaxa Biosystems, Koln, Germany) as recommended by the manufacturer. Thirty-six hours after transfection, cells were screened under 1 mg/mL G418 (Merck, Darmstadt, Germany) for 25 days. Individual G418-resistant colonies were subcloned as Raji/shNC and Raji/shPEBP4 or Daudi/shNC and Daudi/shPEBP4. Raji and Daudi were stably transfected with the hPEBP4-B expression vectors hPEBP4-B or p75PEBP4-B (the PE-binding domain-truncated vector) [19], and with pcDNA3.1/Myc-His (-) B as a mock control. The hPEBP4 expression was confirmed by real-time PCR and Western blot analysis.

### Immunohistochemistry Analysis of hPEBP4 Expression in Human Lymphoma

The human tissue microarrays of lymphoma tissues were obtained from Cybrdi (Xi’an, Shanxi, China). The arrays contain 55 dots in total and each dot represents one normal or diseased tissue spot from one individual specimen that was selected and pathologically confirmed. The arrays were fixed with formalin, embedded in paraffin and immunostained with anti-hPEBP4 antibody using avidin-biotin peroxidase complex method. The results were scored based on the staining intensity which was graded according to the following criteria: - (no staining), +/− (weak and partial staining),+(moderate staining, 30–60% tumor cells), and++(strong staining, >60% tumor cells). Staining index was calculated as the product of staining intensity score of positive tumor cells.

### Cytotoxicity Assays

ADCC activity was measured by lactate dehydrogenase (LDH)-releasing assay using the Cytotoxicity Assay Kit (Cytotoxicity 96 assay for LDH, Promega, USA). CDC activity were measured by LDH-releasing assay or propidium iodide (PI) (Sigma-Aldrich, St. Louis, MO, USA) exclusion assay. For LDH-releasing assay, target cells (Raji and Daudi trasfectants) were preincubated with 20 µg/ml rituximab for 60 minutes at 37°C. Cells were then plated onto 96-well microplates (Costar, Corning Inc., Corning, NY) at 10,000 cells/well. Freshly isolated peripheral blood mononuclear cells (PBMCs) or normal human serum (NHS) was obtained from healthy volunteers. PBMCs were then added at a predetermined effector to target ratio of 50∶1 or 25∶1 for the ADCC assay. NHS (10% vol/vol) as a source of complement was added to target cells for CDC assay at a final volume of 100 µl. After further incubation for 4 hr at 37°C, cell lysis was assessed by measuring the amount of LDH release into the culture supernatant. Maximum (100%) LDH release was measured by addition of 0.5 µl of 1% Triton-X-100 to lyse cells. LDH concentration was assayed on a microtiter plate reader (Bio-RAD680, America) at 490 nm [8]. The percentage of specific lysis was calculated according to the following formula: % lysis = [experimental release-spontaneous release]/[maximum release-spontaneous release]×100. For CDC activity determined by PI exclusion assay, 1×10^6^ target cells (Raji and Daudi trasfectants) were preincubated with 20 µg/ml rituximab for 60 minutes at 37°C, and then incubated with 2% NHS for 60 minutes. Cell death was detected by flow cytometry after PI staining.

### Immunotherapy

Groups of 8-wk-old female BALB/c nude mice (Slac Laboratory Animal, Shanghai, China) were randomized into 4 groups of 9 mice or 12mice. Mice were injected via the tail vein with 5×10^6^ stable transfectants of Raji cells(Raji/shPEBP4 or Raji/shNC cells) or day 0, followed 5 days later by peritoneal injection of 100 µg/per mouse of rituximab or PBS. The mice were examined every 2 days for sign of sickness, and followed until they were sacrificed at onset of hind leg paralysis or up to 90 days. This study was carried out in strict accordance with the recommendations in the guide for the care and use of laboratory animals of the national institutes of health. The protocol was approved by the Committee on the Ethics of Animal Experiments of Zhejiang University Institutional Animal Care and Use Committee. (Permit Number: 2011–11). All efforts were made to minimize suffering.

### Kinetic Studies of ROS Generation and Calcium Flux

For determination of intracellular ROS generation, cells were preincubated with 20 µg/mL rituximab for 5 minutes, and then incubated with 10 µΜ DHE for 15 minutes at 37°C. Cells were excited at 480∼535 nm and emission was measured at 590∼610 nm. To establish a baseline fluorescence for 1 to 2 minutes, unstimulated cells were analyzed by FACS Calibur flow cytometry (Becton-Dickinson, USA). Then, serum was added after the first minute of cell acquisition and ROS production was recorded for 13–15 minutes. For determination of intracellular calcium flux, cells were preincubated with 4 µg/ml Flou4 and 0.4% pluronic-F127 for 1 hr at 37°C. Cells were excited at 490 nm and emission was measured at 520 nm. To establish a baseline fluorescence for 1 to 2 minutes, unstimulated cells were analyzed by FACS Calibur flow cytometry, and then cells were stimulated with 10% NHS and calcium flux was recorded for 6–10 minutes.

### Localization of hPEBP4 Using Spinning Disk Confocal Microscopy (SDCM)

Raji cells were transiently transfected with hPEBP4-GFP, p75PEBP4-GFP, or GFP control vector. After 24 hours, the transfectants were incubated with 10 µM LysoTracker Red DND-99 (Invitrogen Carlsbad, CA USA) for 15 min at room temperature. At the same time, hPEBP4-GFP, p75PEBP4-GFP or mock vector was co-transfected into Raji cells with pDsRed-mem (encoding a membrane-localized form of red fluorescent protein). Twenty-four hours after transfection, the cells were treated with 20 ug/ml rituximab for 1 hr, washed once and then transferred onto a glass bottom dish. The glass bottom dish was placed on a stage prewarmed to 37°C, 10% NHS was added into the media, and cells were analyzed by SDCM immediately. The individual still images were converted by MetaMorph software.

### Proliferation and Cell Viability Assay

Proliferation and cell viability were assessed using the standard Cell Counting Kit-8 (CCK-8) assay kit (Dojindo Molecular Technologies, Gaithersburg, MD, USA) which measures the metabolic activity of viable cells [10]. The cells were treated with 10 µg/ml rituximab or 1 µM CPT for 24 hr, or pretreated with 10 µg/ml rituximab for 24 hr followed by CPT for another 24 hr incubation. DMSO (0.1%) was used as the vehicle control for CPT. The percentage of proliferation and cell viability was calculated using the background corrected readings as follows: Proliferation (%) or cell viability (%) = mean absorbance of sample wells/mean absorbance of untreated cells ×100. Cell viability was determined by measuring the absorbance at 490 nm. Experiments were performed independently in triplicate. The absorbance values obtained from the groups which were treated by different concentrations of CPT with or without rituximab were used to calculate the IC50 of CPT using the Chou’s dose-effect equation as described elsewhere [25].

### Flow Cytometry

Raji cells were stained using FITC-labeled antibodies (BD Pharmingen, San Diego, CA, USA) against human CD20, human CD46, human CD55 and human CD59. B-ALL cells were incubated with antibody against human CD20, fixed with 1% paraformaldehyde and permeabilized with 90% methanol, then incubated with goat anti-hPEBP4 antibody or isotype antibody for 2 h followed by donkey anti-goat IgG antibody (BD Pharmingen, San Diego, CA, USA).

### Statistical Analysis

Statistical analysis was performed by Student’s *t* test to identify significant differences unless otherwise indicated. Differences were considered significant at a *P* value of <0.05. *P* values for differences in survival between treatment and control group were calculated by a log-rank test. For the data obtained from flow cytometry, all data shown in this article were representative of at least three independent experiments.

## Results

### Human Phosphatidylethanolamine-binding Protein 4 is Highly Expressed in Human Lymphoma Tissues

**Figure 1:**
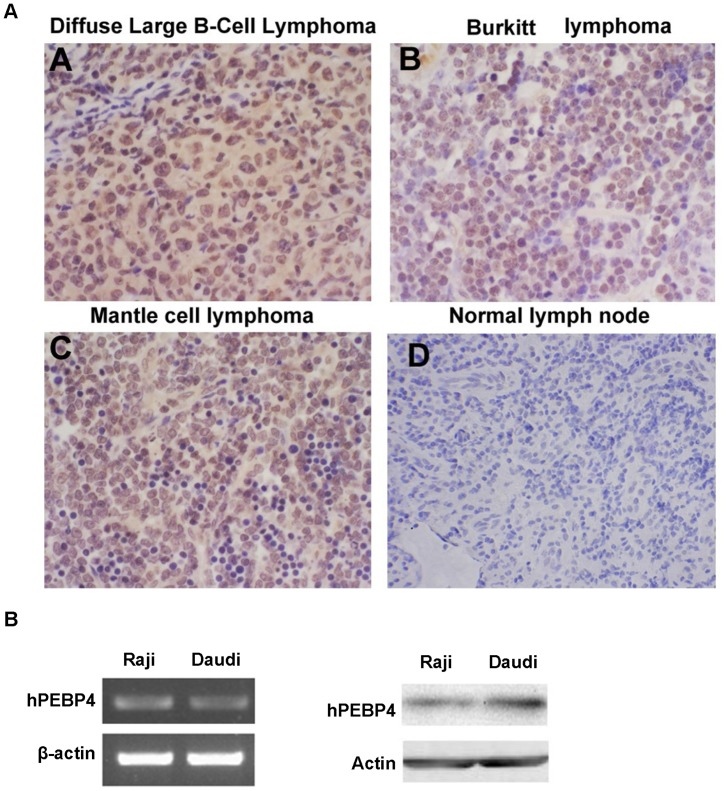
hPEBP4 is highly expressed in human lymphoma. A. Representative results of immunohistochemical staining of hPEBP4 protein (Yellow) in one sample with no signal in the normal lymph node (panel d) but positive staining in lymphoma samples (panels a–c). Photos were taken under×200 magnifications. B. RT-PCR (left) and Western blot analysis (right) of hPEBP4 expression in B-NHL cell line.

hPEBP4 is highly expressed in several solid neoplasms such as human breast cancer, prostate cancer, colorectal cancer and lung cancer [14]–[17], but whether this is true for hematologic malignancies remains undetermined. Hence, we investigated the expression pattern of hPEBP4 in clinical specimens of normal and tumor lymph node tissue using tissue microarrays. In the tissue arrays, we used the standard immunohistochemical protocol and criteria for the judgment of positive or negative signals. As shown in Fig. 1A and Fig. S1, lymphomas including diffuse Large B-cell lymphoma, Burkitt lymphoma, mantle cell lymphoma were positive for hPEBP4 expression. Normal lymph node tissue was essentially negative for hPEBP4 expression. Moreover, hPEBP4 expression was found to be present in almost all the lymphoma cases with 96.7% in B lymphoma samples (29/30), 92% in T lymphoma samples (12/13) and only 16.7% in normal lymph tissue that stained positive (Table 1). The difference in the prevalence of hPEBP4 between lymphoma and normal lymph node was found to be highly significant (P = 0.0001), indicating the preferential expression pattern of hPEBP4 in human lymphoma tissues. We also observed that B non-Hodgkin lymphoma (B-NHL) cells Daudi and Raji expressed high levels of hPEBP4 (Fig. 1B).

**Table 1:** Summary of archival lymphoma samples tested using Immunohistochemistry, showing the percentage of samples positive for hPEBP2.

Tissue type	Total no. studied	Immunohistochemisty positive[no.(%)]
Normal lymph nodes	12	2(16.7)
**B cell lymphoma**	30	29(96.7)*^a^*
Diffuse large B-cell lymphoma	9	8(88.9)
Mantle cell lymphoma	2	2(100)
Follicular Lymphoma	3	3(100)
B-Lymphoblastic leukemia/lymphoma	2	2(100)
Extranodal marginal zone lymphoma MALT lymphoma	7	7(100)
Burkitt lymphoma	4	4(100)
B-chronic lymphocytic leukemia/small lymphocytic leukemia	3	3(100)
**T- cell lymphoma**	13	12(92) *^b^*
Precursor T-cell neoplasm	4	3(75)
Angioimmunoblastic T-cell lymphoma	3	3(100)
Peripheral T-cell lymphoma	6	6(100)

### hPEBP4 Inhibited Rituximab-mediated Complement Dependent Cytotoxicity (R-CDC) and Antibody-dependent Cell-mediated Cytotoxicity (ADCC) in Human Lymphoma Cells

**Figure 2:**
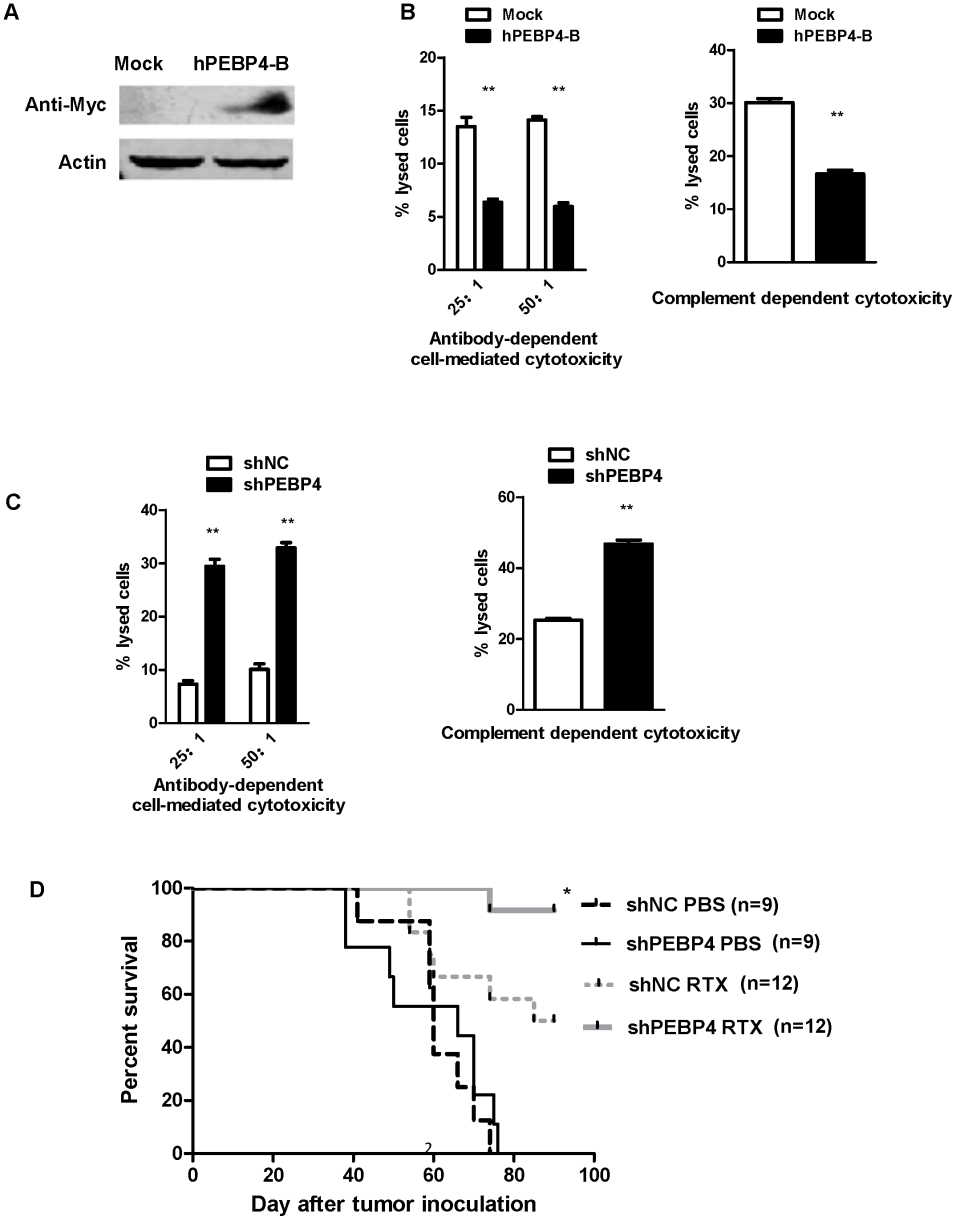
hPEBP4 overexpression inhibited rituximab-mediated ADCC (antibody-dependent cell-mediated cytotoxicity) and CDC (complement-dependent cytotoxicity) in human lymphoma cells. A. Raji cells were stably transfected with hPEBP4-B or mock vectors, and the expression of hPEBP4 was confirmed by Western blot analysis with anti-hPEBP4 antibody. B. Effects of hPEBP4 overexpression on rituximab-mediated ADCC and CDC. The CDC activity in Raji/hPEBP4-B and Raji/Mock using 10% NHS was measured using a standard LDH assay as described in the materials and methods. **, p<0.01 compared with mock transfected cells. C. Silencing of hPEBP4 sensitized Raji cells to rituximab-mediated ADCC and CDC. **, p<0.01 compared with shNC transfected cells. Results are mean±S.E. of three independent experiments. D. Longer survival of Raji/shPEBP4 bearing nude mice than of control groups when treated with rituximab (p<0.05). Representative of two independent experiments.

Rituximab has been successfully employed in the treatment of B-cell lymphoma because of its CDC and ADCC effect [5], [26]. Given that hPEBP4 is anti-apoptotic [15]–[17], [19] and that it is highly expressed in human lymphoma cancer tissue, we questioned whether hPEBP4 plays a role in rituximab activity against lymphoma. B-NHL Raji and Daudi cells were stably transfected with hPEBP4-B (the hPEBP4 expression vector) or control vector. Western blot confirmed hPEBP4 overexpression in Raji stable transfectants (Fig. 2A). Raji/hPEBP4-B cells exhibited growth characteristics similar to Raji/Mock(data not shown). The efficiency of rituximab mediated ADCC and CDC in stable transfectants was assessed by analyzing the percentage of dead cells using a standard LDH assay. A lower level of death was observed in the hPEBP4-B stable transfectants than in the mock transfectants (Fig. 2B, P<0.01 for CDC, P<0.01 for ADCC). Simultaneously, Raji cells were stably transfected with hPEBP4-RNAi or shNC, and the downregulation of hPEBP4 by RNAi was confirmed by real time PCR and western blot (Fig. S2). Accordingly, hPEBP4-silenced Raji cells were more sensitive to rituximab-mediated death than the shNC transfectants (Fig. 2C P<0.01 for CDC, P<0.01 for ADCC), further confirming that hPEBP4 inhibits rituximab-mediated ADCC and CDC in B-NHL Raji cells. The same results were obtained in another B-NHL cell line, Daudi cells (data not shown).

To be able to investigate whether hPEBP4 silencing can potentiate the therapeutic efficacy of rituximab *in vivo*, we conducted subsequent experiments in nude mice bearing systemic Raji tumor cells. Groups of nude mice were injected with Raji/shPEBP4 or Raji/shNC cells, and then were treated with PBS or rituximab. The survival curves were plotted according to Kaplan-Meier method and compared using long-rank test. No statistical difference in survival was observed between Raji/shNC group and Raji/shPEBP4 group when treated with PBS (Fig. 2D P = 0.4841). However, when rituximab was administered to mice at the dose of 100 ug/per mouse, the survival of nude mice bearing Raji/shPEBP4 was prolonged more significantly than that of nude mice bearing Raji/shNC (Fig. 2D P = 0.0232), demonstrating that downregulation of hPEBP4 effectively enhanced the therapeutic efficacy of rituximab *in vivo*.

### Silencing of hPEBP4 Enhanced the Rituximab-mediated Complement Dependent Cytotoxicity (R-CDC) in Primary B-ALL Cells e*x vivo*

**Figure 3:**
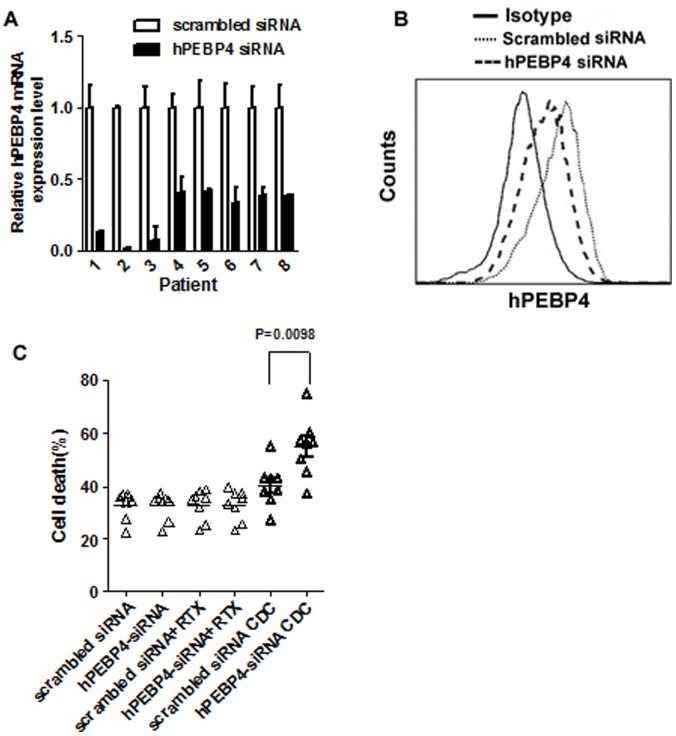
Silencing of hPEBP4 sensitized the primary B-ALL cells to R-CDC effect *ex vivo*. A. 5×10^6^ Primary B-ALL cells from 8 different patients were transfected with hPEBP4 siRNA or scrabble siRNA using the Amaxa nucleofector II (Amaxa Biosystems, Koln, Germany) as recommended by the manufacturer. 24 hours after transfection, Real-time PCR data confirmed the downregulation of hPEBP4. B. Expression of hPEBP4 was determined in B-ALL cells by flow cytometry after intracellular staining. Representative flow cytometry from patient 6, gated on CD20^+^ cells. the purity of the malignant cells were measured by CD20 expression. C-D. R-CDC induced more death in hPEBP4-silent B-ALL cells compared with control (P = 0.0098). 24 hours after transfection, the transfected B-ALL cells were preincubated with 20 µg/mL rituximab for 60 min, and then stimulated by 2% NHS for 1 hr, followed by PI staining.

We showed that downregulation of hPEBP4 increased rituximab-induced complement dependent cytotoxicity in B-NHL cell lines. The clinical relevance of these findings was further investigated using primary B-ALL cells. We selected primary B-ALL cells over B- NHL for its greater accessibility for the analyses. Recently, rituximab has been used clinically in treating CD20^+^ B-ALL as well as in lymphoma [27]. The B-ALL primary cells (90% of cells are CD20 positive, data not shown) obtained from 8 different patients were transfected with hPEPB4 siRNA or scrabble siRNA. Patient characteristics were summarized in the Table S1. Downregulation of hPEPB4 expression using siRNA was confirmed by real-time PCR and flow cytometry (Fig. 3A–B). As shown in Fig. 3C and 3D, B-ALL cells with hPEBP4 downregulation were more sensitive to rituximab-induced death (P = 0.0098). In the B-ALL cells from sample 1 and sample 6, knockdown of PEBP4 using siRNA increased the cell death percentage from 38.2% to 57.2% and 55.0% to 74.9%, respectively, when compared to scrabble siRNA, indicating that hPEBP4 knockdown might act synergistically with rituximab in the treatment of B-cell malignancies.

### Knockdown of hPEBP4 Expression Sensitized the B-NHL Cells to R-CDC Effect by Promoting Calcium Flux and ROS Production

**Figure 4:**
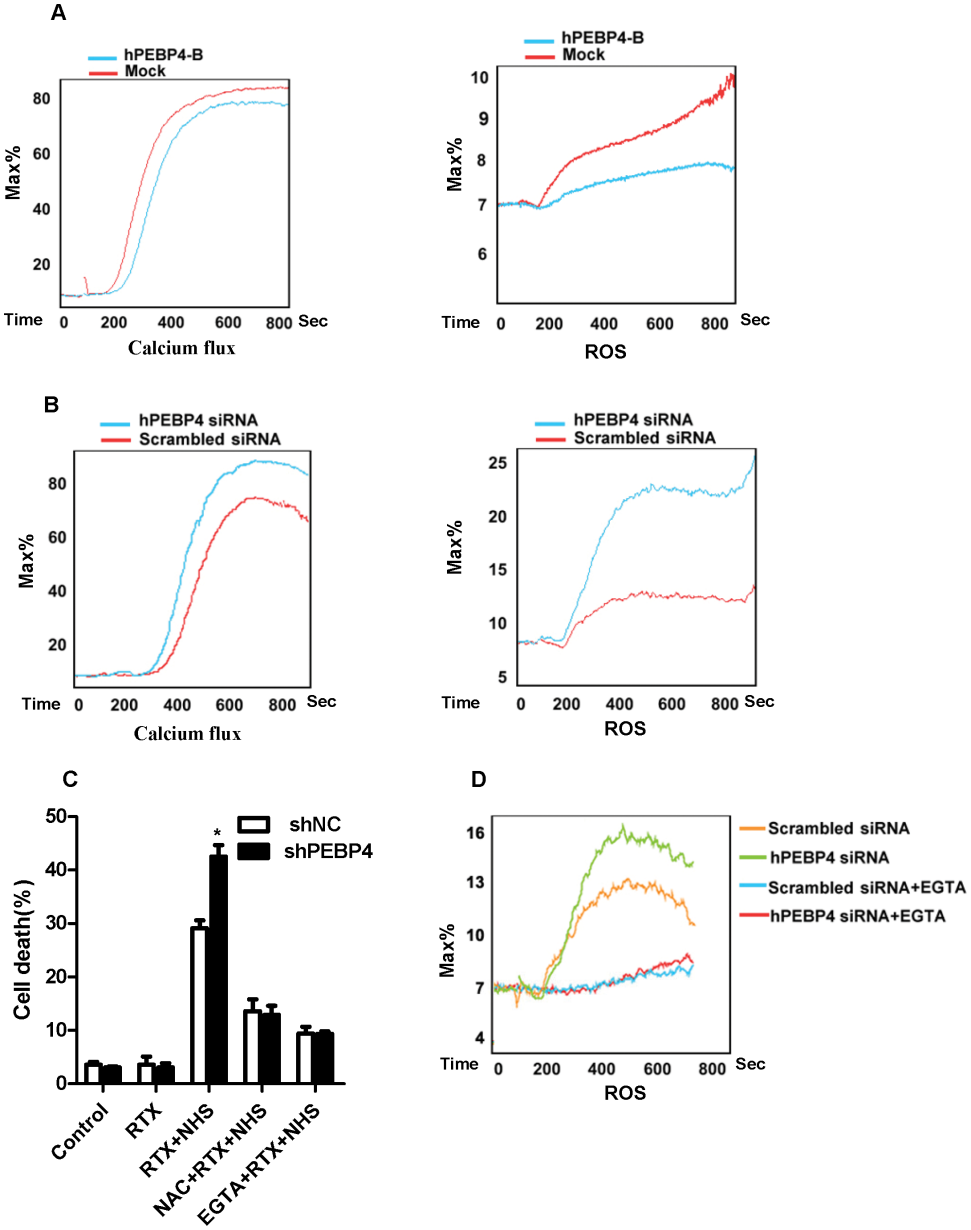
hPEBP4 inhibited the R-CDC effect via blocking Ca2+ flux and ROS production in B-NHL Raji cells. A. Overexpression of hPEBP4 blocked R-CDC induced calcium flux and ROS production in Raji cells. Stable transfectants were preincubated with 20 µg/ml rituximab for 1 hr, before being incubated with DHE or Flou4. Serum was added after the first minute of cell acquisition. B. Time-course of calcium flux and ROS in the Raji cell transfected with hPEBP4 siRNA or scrabble siRNA. C. EGTA or ROS scavenger NAC significantly reversed the potentiating effect of hPEBP4 silencing on R-CDC effect. The stable transfectants of Raji cell were preincubated with EGTA (5 mM) or NAC (50 mM) for 30 min, subsequently treated with 20 µg/ml rituximab for 1 hr, and then stimulated with 2% NHS for 60 min, followed by PI staining. Data are presented as percentage of cell death. Results are mean±S.E of three independent experiments. D. Raji transfectants were preincubated with EGTA (5 mM) or not, the kinetics of ROS generation was detected by FACS analysis.

CD20 has been described as a calcium channel protein. In the presence of a crosslinking antibody, rituximab-mediated death is calcium-dependent [28], [29] whereas R-CDC involves ROS production [6]. Therefore, we investigated whether hPEBP4 influences R-CDC induced calcium flux and ROS production. Addition of 10% NHS in cells preincubated with rituximab (20 µg/ml) resulted in a rapid and intense signal of calcium flux and ROS production. Overexpression of hPEBP4 dampened the R-CDC driven calcium flux and ROS generation (Fig. 4A), while knockdown of hPEBP4 by siRNA resulted in the opposite effect (Fig. 4B **)**. We then investigated whether the observed increase in calcium flux and ROS generation was related to the hPEBP4 knockdown-mediated enhancement of R-CDC effect in B-NHL cells. Raji/shNC and Raji/shPEBP4 cells were incubated with extracellular calcium chelator EGTA or ROS scavenger NAC before addition of rituximab and NHS. EGTA or NAC robustly blocked R-CDC-mediated cell death and reversed the potentiating effect of hPEBP4-knockdown on R-CDC effect (Fig. 4C), indicating that hPEBP4 confers R-CDC resistance in B-NHL cells via inhibiting calcium flux and ROS generation. It is known that a rise in intracellular calcium concentration [Ca^2+^]i results in a higher mitochondrial calcium concentration [Ca^2+^]m, which induces the production of ROS [28]–[30]. In agreement with these studies, EGTA inhibited R-CDC induced ROS production. Furthermore, the increase in ROS production in Raji cells with hPEBP4-knockdown was reversed by EGTA (Fig. 4D). Taken together, hPEBP4 inhibited R-CDC mediated calcium flux, resulting in the impairment of ROS production, which contributes to the R-CDC insensitivity.

### R-CDC Promotes hPEBP4 Translocation to Membrane in B-NHL Cells

**Figure 5:**
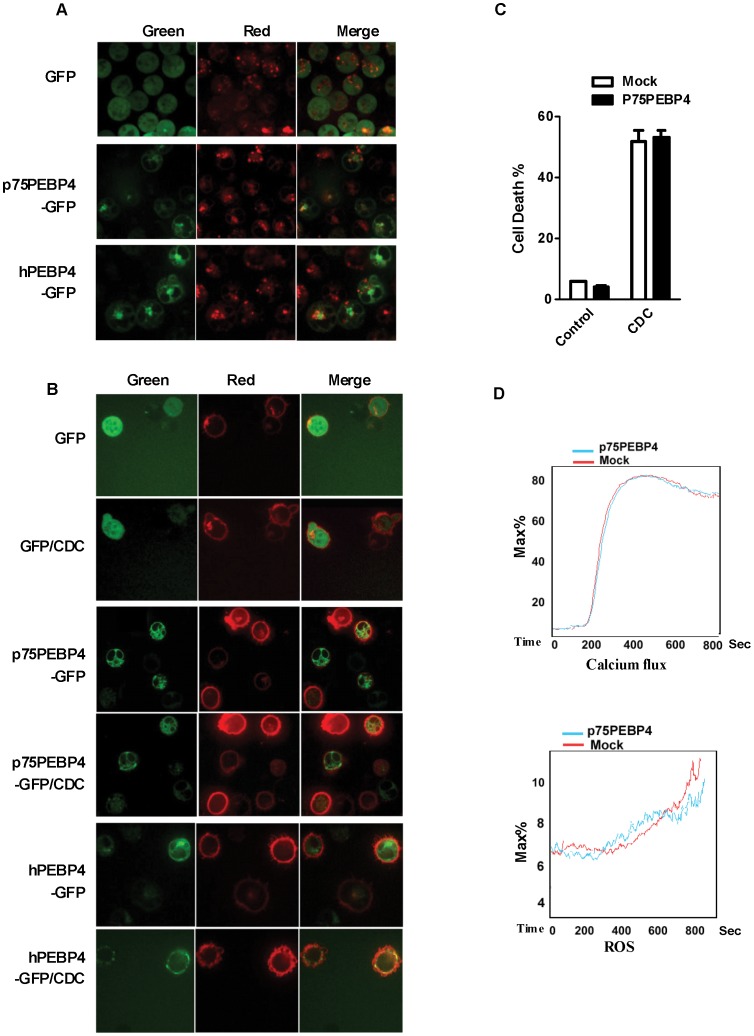
R-CDC promoted hPEBP4 translocation to membrane in B-NHL cells. A. Raji cells were transiently transfected with hPEBP4-GFP, p75PEBP4-GFP or control GFP vector, and 24 hr after transfection, the cells were stained with lysosome-specific LysoTracker Red DND-99. Co-localization of hPEBP4-GFP (green) and lysosomes (red) is demonstrated by yellow fluorescence in overlay images. B. Raji cells were transiently transfected with hPEBP4-GFP, p75PEBP4-GFP or control GFP vector, together with pDsRed-mem. 24 hr after transfection, the cells were opsonized with 20 µg/ml rituximab for 1 hr, and then reacted with 10% NHS for 10 min. Original magnification ×400. C. Overexpression of p75PEBP4 had no effect on rituximab- mediated CDC in Raji cells. Raji cells were transiently tranfected with p75PEBP4-B or mock. The transfectants were pretreated with 20 µg/ml rituximab for 1 hr, then stimulated by 2% NHS for 1 hr, followed by PI staining. D. p75PEBP4 overexpression did not affect R-CDC induced calcium flux and ROS production.

Previous work has shown that anti-CD20 mAbs are capable of inducing a range of intracellular signaling into target cells including Syk, PI3K, p38 and ERK1/2, and these signaling pathways lead to calcium flux following CD20 stimulation [28], [31], [32]. We showed that hPEBP4 confers R-CDC resistance and that this resistance occurs upstream or at the level of calcium flux regulation. We then investigated the mechanism by which hPEBP4 regulates calcium flux. Though knockdown of hPEBP4 enhanced R-CDC induced P38, ERK1/2 and Syk activation (Fig. S3A), the specific inhibitor of P38, MEK1 and Syk not only failed to reverse this enhancement, but also failed to inhibit R-CDC. This observation indicates that P38, ERK1/2 and Syk signaling pathways are not responsible for R-CDC induced cell death (Fig. S3B). We also measured the protein expression of CD20 and of complement regulatory proteins including CD46, CD55 and CD59 in the hPEBP4-silenced Raji cells, and found that the expression levels of these membrane proteins remained almost unchanged. This excluded the possibility that hPEBP4 regulates R-CDC sensitivity by affecting the expression of CD20 and complement regulatory proteins (Fig. S3C). It was previously observed that hPEBP4 could translocate from lysosome to membrane to maintain phospholipid asymmetry, and this protects cells from TNF-α induced-apoptosis [19]. The Raji cells were transfected with hPEBP4-GFP, p75PEBP-GFP (the PE-binding domain-truncated vector), or GFP plasmid, with or without pDsRed-Mem, which encoded a membrane-localized form of red fluorescent protein. Transfected cells were examined by fluorescence confocal microscopy. As shown in Fig. 5A, the majority of hPEBP4-GFP and p75PEBP-GFP green staining overlapped with the red lysosome signal (LysoTracker), as previously reported [19]. The transfected cells were pretreated with rituximab for 60 min, and then stimulated with 10% NHS for 10 min. The real-time changes in fluorescent signals were detected by Spinning disk confocal microscopy (SDCM), As shown in Fig. 5B and Fig. S3D, hPEBP4 translocated to the cell membrane when stimulated by R-CDC. In contrast, GFP, and p75PEBP4-GFP which lacked the PE-binding domain, did not show translocation to the cell membrane. Meanwhile, overexpression of p75PEBP4-B could not induce Raji cells to be refractory to R-CDC induced death (Fig. 5C). Also, p75PEBP4 had no effect on R-CDC induced calcium flux and ROS generation (Fig. 5D). Thus, we showed that hPEBP4 transferred to the plasma membrane in a PE-binding domain dependent manner. This possibly confers resistance to membrane attack complex (MAC), resulting in the impairment of calcium flux and ROS production, which contributes to the insensitivity to R-CDC induced death.

### Silencing of hPEBP4 Potentiated the Chemosensitization of Rituximab in Human Lymphoma Cells

**Figure 6:**
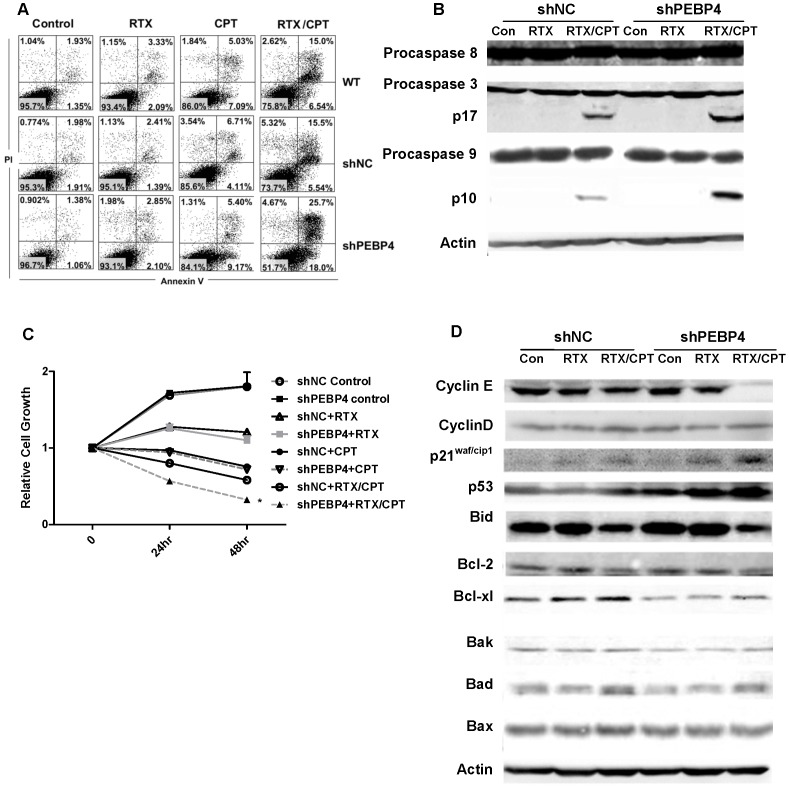
hPEBP4 knockdown sensitized B-NHL cells to rituximab/CPT-induced apoptosis and growth inhibition. A. hPEPB4 stably-silenced Raji cells were pretreated with 10 µg/ml rituximab (RTX) or not for 24 hr, and then treated with CPT (1 µM) for 24 hr. Cells were stained with annexin V/PI and analyzed by flow cytometry. B. Cell lysates were subjected to Western blot analysis and probed with anti-procaspase-8, anti-procaspase-3, anti-procaspase-9. C. Silencing of hPEBP4 enhanced the rituximab/CPT-induced growth inhibition when compared with Raji/shNC (P<0.05). Raji/shNC and Raji/shPEBP4 cells were treated with rituximab (10 µg/mL), CPT (1 µM) or rituximab/CPT, and cell proliferation was detected by CCK-8 assay. The values are expressed relative to 0 hr respectively, which are normalized as 1. The values are mean±S.E. of three independent experiments. D. Examination of a panel of cell cycle-related proteins and apoptosis-related proteins after exposure to rituximab or rituximab/CPT.

A previous study showed that rituximab chemosensitizes B-NHL cells via modifying the signaling pathway involved in apoptosis and cell cycle arrest [33]–[36]. We therefore investigated whether hPEBP4 affects the chemosensitization of rituximab. Fluorescein isothiocyanate-conjugated annexin V/PI staining was performed to detect apoptotic cells. Apoptotic cells are annexin V-positive (including PI- or PI+). In agreement with a previous study, rituximab alone failed to induce cellular apoptosis in B-NHL Raji cells (Fig. 6A). shPEBP4 transfectants exhibited a similar level of apoptosis following treatment with rituximab or CPT, which was widely used in lymphoma treatment [37], [38]. However, these cells exhibited a higher level of apoptosis following the treatment of rituximab in combination with CPT (rituximab/CPT) when compared with shNC transfectants or parental Raji cells (Fig. 6A. Fig. S4A). Moreover, the cytotoxic effect of the rituximab/CPT became progressively more evident between shPEBP4 transfectants versus shNC transfectants, reaching a statistical difference with 0.1 µΜ CPT (Fig. S4B). Accordingly, overexpression of hPEBP4 blocked rituximab/CPT induced-cellular apoptosis (Fig. S4C). The same result was observed in B-NHL Daudi cells (data not shown). hPEBP4 silencing had no effect on rituximab/CPT-induced caspase-8 activation. However, more activated caspase-3 and caspase-9 were were detected in the Raji/shPEBP4 cells following rituximab/CPT treatment (Fig. 6B). This is in agreement with our previous result that hPEBP4-mediated TRAIL resistance occurs downstream of caspase-8 [17]. Raji/shPEBP4 cells exhibited growth characteristics similar to Raji/shNC cells at its baseline level or with the treatment of rituximab or CPT alone. However, they were more sensitive to rituximab/CPT-induced cell growth arrest (Fig. 6C). Meanwhile, we performed the CCK-8 assay to determine viability of cells with hPEBP4 knocked down at different CPT concentrations to further confirm that loss of hPEBP4 enhances the chemosensitization of rituximab. In the presence of rituximab, the IC50 of CPT dropped by almost one seventh from 398.11 µΜ in shNC transfectants to 77.62 µΜ in Raji/shPEBP4 cells and from 7.94 µΜ to 1.07 µΜ in Daudi transfectants (Table S2). However, loss of hPEBP4 did not affect the IC50 of CPT without rituximab. These data clearly show that silencing of hPEBP4 potentiates the chemosensitization of rituximab, and makes B-NHL cells more susceptible to the cytotoxic effect of the chemotherapy agent CPT when administered in combination with rituximab. We then analyzed the cell cycle kinetics of hPEBP4-silenced Raji cells. Representative cell cycle profiles of transfected Raji cells are shown as histograms with data expressed as mean percentage of cells in each cell cycle phase 24 hours after treatment (Fig. S3D). After rituximab/CPT treatment, Raji/shPEBP4 cells showed a higher proportion of cells at G0–G1 phase (69.02%) compared with Raji/shNC control (54.74%) and a decrease in the proportion of cells it S phase (19.75% versus 31.14% ). No difference in the cell cycle profile was found upon rituximab exposure. Given that hPEBP4 not only inhibits rituximab-induced death but also its chemosensitization, silencing of hPEBP4 may be a promising approach in the treatment of B lymphoma due to its synergistic effect with rituximab therapeutic efficacy.

Because the appropriate temporal activation of cyclin E/cyclin-dependent kinase 2 and cyclin D1/cyclin-dependent kinase 2 is required for progression through the G1 and S entry, we monitored the expression of cyclin E, cyclin D1 and the negative regulators of cell cycle progression p21^waf/cip1^ in Raji/hPEBP4-silenced cells. Interestingly, cyclin E expression level was significantly decreased in Raji/hPEBP4 cells when compared with Raj/shNC after rituximab/CPT treatment, whereas cyclin D1 expression level did not differ (Fig. 6D). On the other hand, hPEBP4 knockdown enhanced rituximab/CPT-induced p21^waf/cip1^expression. In addition, hPEBP4 knockdown significantly promoted p53 expression and downregulated Bcl-xl expression in Raji cells while the expression level of other apoptosis-related molecules remained unchanged.

## Discussion

Rituximab has been used for lymphoma immunotherapy during the recent two decades and has become the standard regimen to treat B-NHL alone or in combination with chemotherapy. However, 30–40% of B-NHL patients are unresponsive to rituximab or resistant after therapy [39]. Therefore, novel treatment regimens in which rituximab are combined with therapeutics that interfere with resistance mechanisms are needed. Interestingly, hPEBP4, a novel member of the human PEBP family that we identified, is selectively expressed in several types of cancer cells and functions as anti-apoptotic molecule. Downregulation of endogenous hPEBP4 in breast cancer cells sensitized cells to TNF-α-induced apoptosis and cell cycle arrest. In prostate cancer cell lines, hPEBP4 expression negatively correlates with sensitivity to TRAIL-induced apoptosis [15], [17]. Here, we demonstrated that hEPBP4 renders the B-NHL Raji and Daudi cells resistant to rituximab activity. Silencing of hPEPB4 promoted sensitivity of B-NHL cells and primary B-ALL cells to rituximab-induced complement dependent cytotoxicity and augmented the rituximab therapeutic efficacy *in vivo.*

In an effort to enhance the efficacy of rituximab-induced immunotherapy in B-NHL, a variety of methods, such as mAb, siRNA and peptide treatments, have been used to neutralize or decrease the expression of some molecules related to rituximab resistance, such as CD59 [11]. However, these treatments often produce multiple side effects because they target both tumor cells and normal cells. In our study, the tissue microarray examination of human lymphoma showed that almost all of the lymphoma tissues express hPEPB4, but normal lymph nodes rarely express hPEBP4. The specific abundance of hPEBP4 in lymphoma tissues and its ability to confer rituximab resistance strongly imply that hPEBP4 could be a potential target for knockdown in the treatment of B-NHL when administered in combination with rituximab.

Rituximab contains a human Fc of the isotype IgG1. Hence, the bound antibody labels CD20-positive cells for recognition by the complement system and by constant antibody fragment receptor (FcR)-bearing immune cells. During ADCC, immune effector cells such as natural killer (NK) cells, macrophages and neutrophils, recognize the Fc of rituximab via their FcR. This leads to release of perforin, granzymes and tumor necrosis factor (TNF), which can induce target cell death [4]. hPEBP4 was previously identified to inhibit TNF-α induced apoptosis, which may contribute to resistance to ADCC in hPEBP4 overexpressing B-NHL cells.

To understand the mechanisms by which hPEBP4 confers rituximab-mediated complement dependent cytotoxicity(R-CDC) resistance, we evaluated the role of hPEBP4 on calcium flux and ROS production which was reported to be involved in anti-CD20 mAb-induced death [6], [29], [40]. hPEBP4 significantly blocked R-CDC-induced calcium flux and ROS generation. Calcium chelator EGTA or ROS scavenger NAC reversed the potentiating effect of hPEBP4-silencing on R-CDC-induced cell death. Meanwhile, EGTA restored the increase in ROS production resulting from hPEBP4 knockdown in B-NHL cells. Thus, hPEBP4-mediated regulation of calcium flux and ROS generation appears to be responsible for the observed inhibition of R-CDC effect, and this inhibition occurs upstream or at the level of calcium flux regulation.

Penetration of antibody-opsonized cells by membrane attack complex (MAC) of complement promotes calcium flux [40]. Our previous study showed that hPEBP4 translocated from lysosomes to membrane upon TNF-α stimulation to maintain the phospholipid asymmetry [19]. In this present study, we found R-CDC induced hPEBP4 to translocate to the plasma membrane in a PE-binding domain dependent manner. In accordance with this result, p75PEBP4-B which lacked the PE-binding domain did not affect R-CDC-induced calcium flux and ROS generation in B-NHL cells, thus losing its inhibitory effect on R-CDC induced death. In summary, these findings suggest that hPEBP4 translocates to the plasma membrane, binds with PE, and confers resistance to MAC attack. These events possibly result in the impairment of calcium flux and ROS production, which contributes to the cells insensitivity to R-CDC induced death.

In addition to inducing ADCC and CDC, rituximab interferes with the intracellular signal transduction pathway and sensitizes B-NHL cell lines to chemotherapeutic drugs via selective down-regulation of Bcl-2 and Bcl-xl [13], [33]. hPEBP4 inhibited rituximab/CPT-induced apoptosis and growth inhibition in B-NHL cell lines, but had no effect on rituximab or CPT alone induced apoptosis and growth inhibition. We also showed that only in the presence of rituximab that loss of hPEBP4 favored the cytotoxicity of CPT and caused almost 7-fold decrease of IC50 in B-NHL cells, clearly confirming that silencing of hPEBP4 enhances chemosensitization of rituximab in B-NHL cells. Meanwhile, loss of hPEBP4 expression diminished the expression of Bcl-xl and cyclin E, and significantly increased the p53, p21^waf/cip1^ proteins level. Thus, hPEBP4 silencing may augment the ability of rituximab to inhibit the survival pathway activation, thus potentiating its chemosensitizing effect in the B-NHL cells.

As a member of PEBP family, hPEBP4 has a PE binding domain just like RKIP (PEBP1). However they seem to play distant roles in apoptosis and cancer invasion. RKIP/PEBP1 is pro-apoptotic [41]–[43] while hPEBP4 is anti-apoptotic[15]–[17], [19]–[20]. Recently, RKIP has been implicated as a metastatic suppressor for prostate cancer and breast cancer [44], [45] while hPEBP4 overexprression positively corelated with the metastasis of lung squamous cell carcinomas and colorectal cancer [14], [18]. We also found silencing of hPEBP4 enhanced the the RKIP expression in the Raji cells (data now shown), indicating the regulation between the expression of RKIP and hPEBP4 via unknown mechanisms. It is also possible that the non-conserved domain is responsible for the different function of RKIP and hPEBP4 on apoptosis. Further nvestigation will be required to clarify the exact mechanisms account for the different function of hPEBP4 and RKIP/PEBP1 on the apoptosis.

Taken together, we propose that hPEBP4 plays an important role both in the direct and indirect effects of rituximab. Given the preferential expression pattern of hPEBP4 in lymphoma tissues, hPEBP4 may be a potential target for therapeutic intervention in the treatment of lymphoma combined with rituximab. IOI-42, as a novel small molecule inhibitor of hPEBP4, has been reported to sensitize tumor cells to TNF-α and TRAIL-mediated apoptosis [20], suggesting that hPEBP4 interference with a specific pharmacologic inhibitor is capable of sensitizing cancer cells when used in combination with other drugs. Investigating whether IOI-42 is able to potentiate the rituximab therapeutic efficacy will further clarify the clinical ramifications of the therapeutic targeting of hPEBP4 in lymphoma and may provide a new avenue for the clinical application of rituximab.

## Supporting Information

Figure S1 Immunohistochemical staining of isotype control in human lymphoma.
(JPG)

Figure S2 Stable silencing of hPEBP4 expression in Raji cells was confirmed by real-time PCR (A) and Western blot analysis (B).
(JPG)

Figure S3 **R-CDC drives hPEBP4 translocation to membrane in B-NHL cells.** A.Raji/shNC and Raji/shPEBP4 cells were pretreated with rituximab (20 µg/mL) for 1 hr, and then incubated with 10% NHS for various times, equivalent protein loadings of each lysate were immunoblotted with antibodies recognizing the phosphorylated, active forms of p42/44 ERK1/2, Syc and p38. B. ERK1/2, Syk, and p38 inhibitors fail to reverse the potentiating effect of hPEBP4 silencing in rituximab mediated CDC. The stable transfectants of Raji and Daudi cells were preincubated with Syk inhibitor (PP2), MEK inhibitor (U0126), p38 inhibitor (SB203580) (all at 10 uM) or DMSO (0.1%) for 20 min, subsequently treated with 20 µg/ml rituximab for 1 hr, and then stimulated with 2% NHS for 60 min, followed by PI staining. Representative of three independent experiments. C. hPEBP4 RNA interference does not affect the surface expression levels CD20, CD46, CD55, CD59. Representative of three independent experiments. D, Raji cells were transiently transfected with hPEBP4-GFP, p75PEBP4-GFP or control GFP vector, together with pDsRed-mem. 24 hr after transfection, the cells were opsonization with 20 µg/ml rituximab for 1 hr, and then reacted with 10% NHS for 10 min. Original magnification ×400.
(JPG)

Figure S4 **hPEBP4 inhibits rituximab/CPT-induced apoptosis in B-NHL cells.** A. The stable transfectants of Raji cells were treated with CPT (1 µM) at various times, following incubation with rituximab for 24 hr. B. Loss of hPEBP4 significantly enhances rituximab/CPT-induced apoptosis in B-NHL cells. ***, *p<*0.001 compared with shNC transfectants, experiments performed in quadruplicate, means±S.E. C. Overexpression of hPEBP4 confers resistance to rituximab/CPT-induced apoptosis. Raji cells stably transfected with hPEBP4-B or mock were pretreated with 10 µg/mL rituximab or not, and then treated with CPT (1 µM) for 24 hr, followed by FACS analysis for apoptosis assay. D. hPEBP4 silencing increases rituximab/CPT induced G0–G1arrest in Raji cells. Stably transfected Raji cells were treated as described in apoptosis assay, and PI staining was used to analyze cell cycle distribution. Representative of three independent experiments.
(JPG)

Table S1 **Patient characteristics.** Patient characteristics including gender, age, mutation of BCR/ABL, genetic aberrations, expression of CD20, and previous therapy.
(DOCX)

Table S2 **Cytotoxic effects of CPT alone or CPT in the presence of rituximab on cell viability of shNC and shPEBP4 transfectants at 24** **hr as determined by the CCK-8 assay.**
(DOCX)
