# Supplementary figures and images for: Silencing of Human Phosphatidylethanolamine-Binding Protein 4 Enhances Rituximab-Induced Death and Chemosensitization in B-Cell Lymphoma
Source: PLoS One. 2013 Feb 25;8(2):e56829. doi: 10.1371/journal.pone.0056829 (PMC3581549; doi:10.1371/journal.pone.0056829)

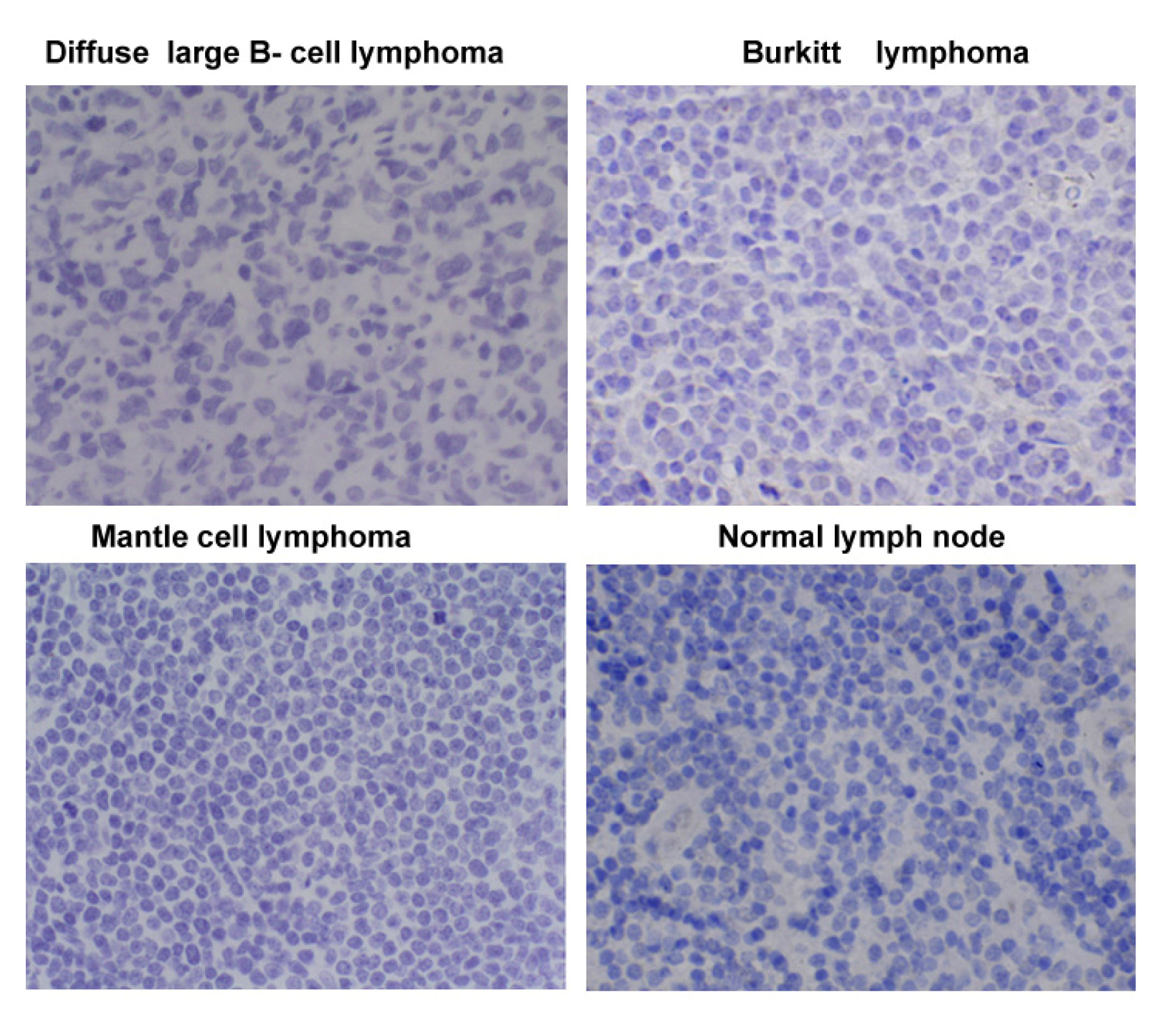

Supplement: Figure S1 — Immunohistochemical staining of isotype control in human lymphoma. (JPG) [file pone.0056829.s001.jpg]

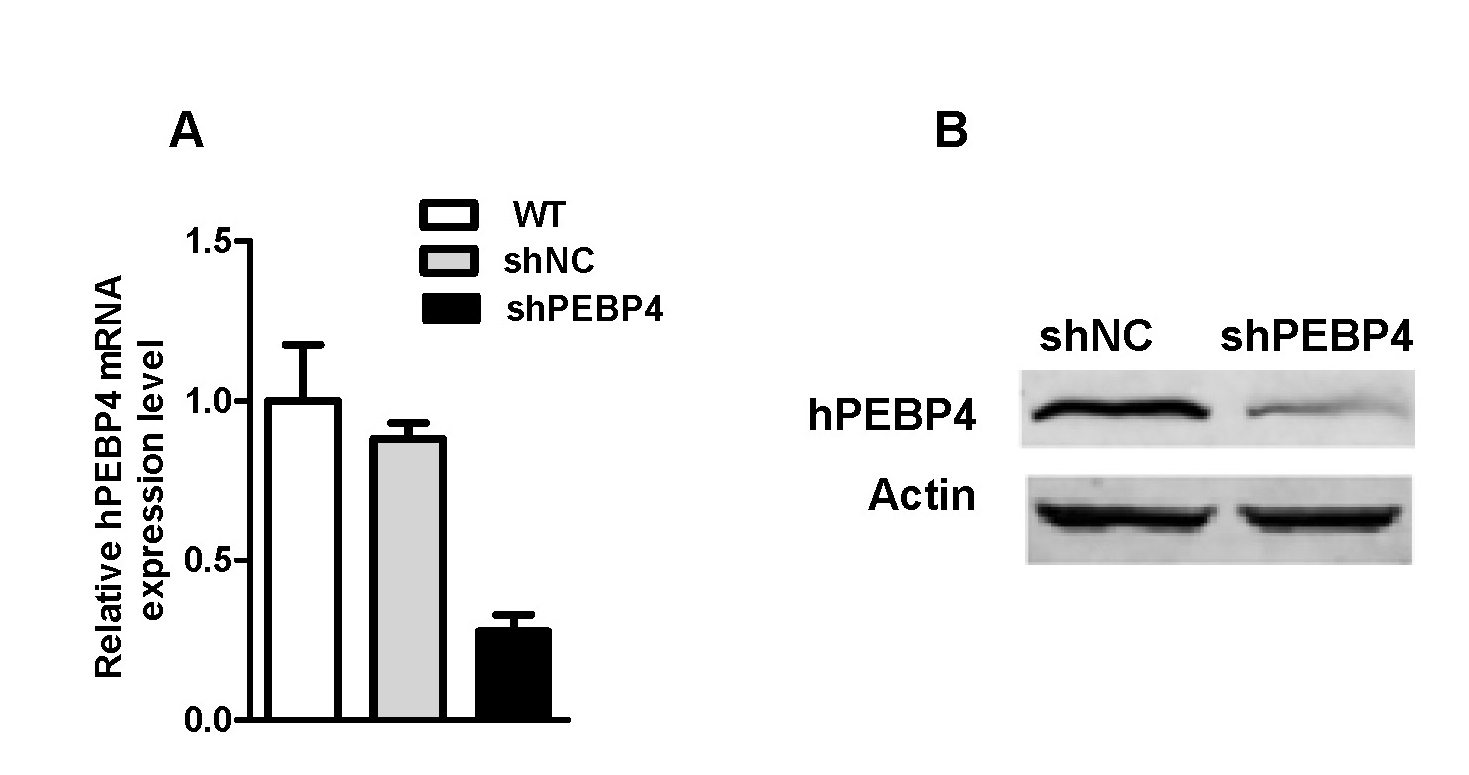

Supplement: Figure S2 — Stable silencing of hPEBP4 expression in Raji cells was confirmed by real-time PCR (A) and Western blot analysis (B). (JPG) [file pone.0056829.s002.jpg]

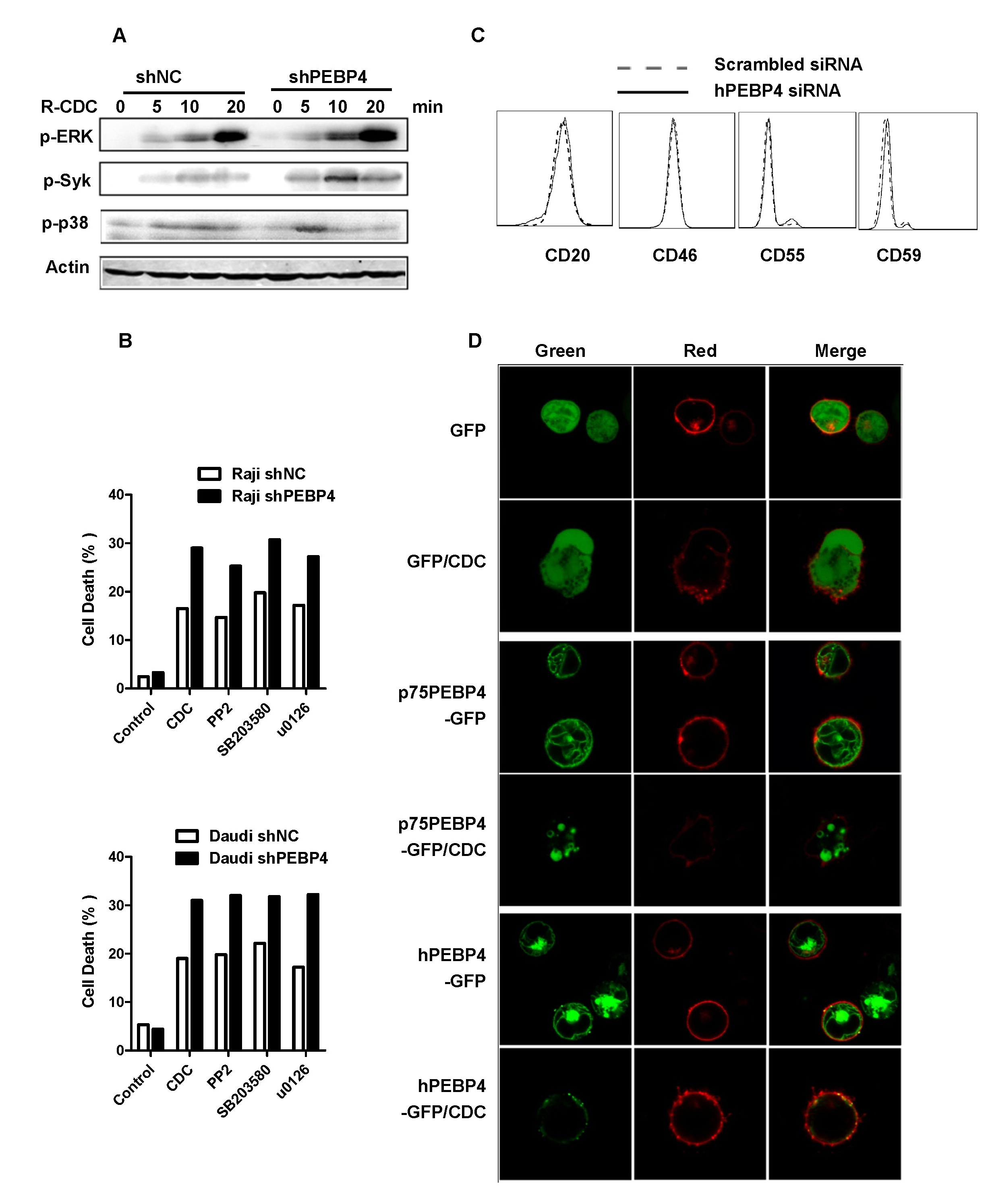

Supplement: Figure S3 — R-CDC drives hPEBP4 translocation to membrane in B-NHL cells. A.Raji/shNC and Raji/shPEBP4 cells were pretreated with rituximab (20 µg/mL) for 1 hr, and then incubated with 10% NHS for various times, equivalent protein loadings of each lysate were immunoblotted with antibodies recognizing the phosphorylated, active forms of p42/44 ERK1/2, Syc and p38. B. ERK1/2, Syk, and p38 inhibitors fail to reverse the potentiating effect of hPEBP4 silencing in rituximab mediated CDC. The stable transfectants of Raji and Daudi cells were preincubated with Syk inhibitor (PP2), MEK inhibitor (U0126), p38 inhibitor (SB203580) (all at 10 uM) or DMSO (0.1%) for 20 min, subsequently treated with 20 µg/ml rituximab for 1 hr, and then stimulated with 2% NHS for 60 min, followed by PI staining. Representative of three independent experiments. C. hPEBP4 RNA interference does not affect the surface expression levels CD20, CD46, CD55, CD59. Representative of three independent experiments. D, Raji cells were transiently transfected with hPEBP4-GFP, p75PEBP4-GFP or control GFP vector, together with pDsRed-mem. 24 hr after transfection, the cells were opsonization with 20 µg/ml rituximab for 1 hr, and then reacted with 10% NHS for 10 min. Original magnification ×400. (JPG) [file pone.0056829.s003.jpg]

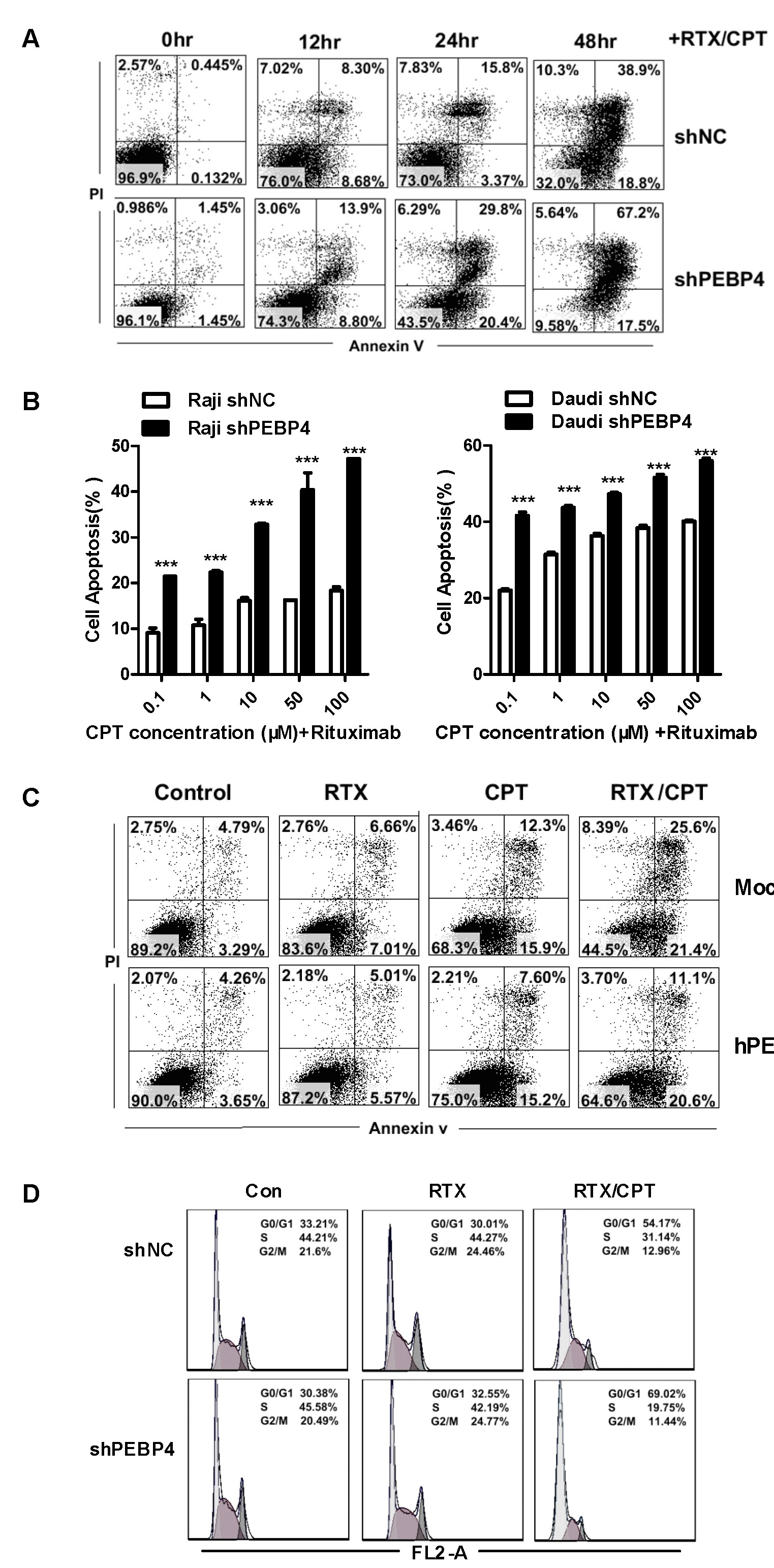

Supplement: Figure S4 — hPEBP4 inhibits rituximab/CPT-induced apoptosis in B-NHL cells. A. The stable transfectants of Raji cells were treated with CPT (1 µM) at various times, following incubation with rituximab for 24 hr. B. Loss of hPEBP4 significantly enhances rituximab/CPT-induced apoptosis in B-NHL cells. ***, p<0.001 compared with shNC transfectants, experiments performed in quadruplicate, means±S.E. C. Overexpression of hPEBP4 confers resistance to rituximab/CPT-induced apoptosis. Raji cells stably transfected with hPEBP4-B or mock were pretreated with 10 µg/mL rituximab or not, and then treated with CPT (1 µM) for 24 hr, followed by FACS analysis for apoptosis assay. D. hPEBP4 silencing increases rituximab/CPT induced G0–G1arrest in Raji cells. Stably transfected Raji cells were treated as described in apoptosis assay, and PI staining was used to analyze cell cycle distribution. Representative of three independent experiments. (JPG) [file pone.0056829.s004.jpg]
