# Supplementary material for: Silencing of Human Phosphatidylethanolamine-Binding Protein 4 Enhances Rituximab-Induced Death and Chemosensitization in B-Cell Lymphoma
Source: PLoS One. 2013 Feb 25;8(2):e56829. doi: 10.1371/journal.pone.0056829 (PMC3581549; doi:10.1371/journal.pone.0056829)
Supplement: Supplementary file 2 [file pone.0056829.s005.docx]

Patient characteristics

Patients	Sex	Age	Bcr/Abl	Genetic aberration	CD20	Previous therapy
1	F	17	-	None	+	None
2	F	29	-	None	+	VDCP
3	M	25	+	t(9;22)(q34,q11),del(9)(p13)	+	VDCP
4	F	16	-	del(1)(p32)	+	VDLD
5	M	44	-	None	+	VDLP
6	M	30	-	None	+	None
7	F	36	+	None	+	VDLD
8	F	31	-	None	+	VICP

F	17			+	VDCP
F	29	-	None	+	VDCP
M	25	+	t(9;22)(q34,q11),del(9)(p13)	+	VLDL
F	16		del(1)(p32)	+	VDLP
M	44	-	None	+	VDLP
M	30	-		+	
F	36	+	None	+	VDLD
F	31	-	None	+	VICP

Abbreviations: VDCP, vindesine + daunorubicin +cytoxan+ dexamethasone; VDLD, vindesine + daunorubicin +L-asparaginase+ dexamethasone; VDLP, vindesine + daunorubicin + L-asparaginase + dexamethasone; VICP, vindesine + idarubicin + cytoxan + dexamethasone; F, female; M, male; +(in the column 'Bcr/Abl') indicated Bcr/Abl fusion gene was detected, - indicated no Bcr/Abl fusion gene; +(in the column 'CD20'), indicated the expression of CD20.

Patient characteristics including sex, age, Bcr/Abl fusion gene, genetic aberration, expression of CD20 and previous therapy.
