# Supplementary material for: Silencing of Human Phosphatidylethanolamine-Binding Protein 4 Enhances Rituximab-Induced Death and Chemosensitization in B-Cell Lymphoma
Source: PLoS One. 2013 Feb 25;8(2):e56829. doi: 10.1371/journal.pone.0056829 (PMC3581549; doi:10.1371/journal.pone.0056829)
Supplement: Supplementary file 3 [file pone.0056829.s006.docx]

hPEBP4 promotes the chemosensitization of rituximab in B-NHL cells
